# Supplementary material for: Low Serum Magnesium is Associated with Incident Dementia in the ARIC-NCS Cohort
Source: Nutrients. 2020 Oct 9;12(10):3074. doi: 10.3390/nu12103074 (PMC7600951; doi:10.3390/nu12103074)
Supplement: Supplementary file 1 [file nutrients-12-03074-s001.zip › Supplemental Figure S1.docx]

**Supplemental Figure 1: Diagram of analytic cohort selection for visits 5 through 7, using visit 5 as baseline.**
