# Supplementary material for: Low Serum Magnesium is Associated with Incident Dementia in the ARIC-NCS Cohort
Source: Nutrients. 2020 Oct 9;12(10):3074. doi: 10.3390/nu12103074 (PMC7600951; doi:10.3390/nu12103074)
Supplement: Supplementary file 1 [file nutrients-12-03074-s001.zip › Supplemental Table S1.docx]

**Supplemental Table 1. Association of visit 5 magnesium quintiles with incident dementia up through visit 7, ARIC 2011-2019.**

| **Quintile of Magnesium** | **Person Years of Follow-Up** | **Number developing dementia** | **IR‡** | **Model 1* HR** | **Model 2** HR** |
| --- | --- | --- | --- | --- | --- |
| **Quintile 1** | 3,559 | 101 | 28.38 | 0.98 (0.75, 1.27) | 0.95 (0.72, 1.26) |
| **Quintile 2** | 3,651 | 97 | 26.57 | 0.94 (0.72, 1.23) | 0.92 (0.70, 1.21) |
| **Quintile 3** | 4,618 | 117 | 25.34 | 0.91 (0.71, 1.17) | 0.91 (0.71, 1.18) |
| **Quintile 4** | 5,014 | 128 | 25.53 | 0.94 (0.73, 1.20) | 0.97 (0.75, 1.24) |
| **Quintile 5** | 4,856 | 127 | 26.15 | 1 (Ref) | 1 (Ref) |
| **Per 1 standard deviation decrease in Mg** | | |  | 1.00 (0.92, 1.08) | 0.98 (0.90, 1.07) |

‡Crude incidence rate, per 1000 person-years.

* Cox proportional hazards model adjusted for age, interaction of age with time, race-center, sex, and education.

**Adjusted for Model 1 variables, plus history of smoking, drinking status, waist-to-hip ratio, western and prudent diet scores, estimated glomerular filtration rate, c-reactive protein, sodium, potassium, calcium, prevalent coronary heart disease, previous stroke, systolic and diastolic blood pressure, antihypertensive diuretic medication use, total-cholesterol-to-HDL cholesterol ratio, diabetes status, and apolipoprotein E4 allele.
