# Supplementary material for: Low Serum Magnesium is Associated with Incident Dementia in the ARIC-NCS Cohort
Source: Nutrients. 2020 Oct 9;12(10):3074. doi: 10.3390/nu12103074 (PMC7600951; doi:10.3390/nu12103074)
Supplement: Supplementary file 1 [file nutrients-12-03074-s001.zip › Supplemental Table S2.docx]

**Supplemental Table 2. Association of baseline magnesium quintiles with incident dementia, stratified by race, ARIC 1990-2019.**

| **Race** | **Quintile of Magnesium** | **Person Years of Follow-Up** | **Number developing dementia** | **IR‡** | **Model 1* HR** | **Model 2** HR** |
| --- | --- | --- | --- | --- | --- | --- |
| **White** |  |  |  |  |  |  |
|  | **Quintile 1** | 18,658 | 197 | 10.56 | 1.34 (1.12, 1.59) | 1.20 (1.00, 1.44) |
|  | **Quintile 2** | 35,557 | 307 | 8.63 | 1.08 (0.92, 1.26) | 1.06 (0.91, 1.24) |
|  | **Quintile 3** | 55,872 | 502 | 8.98 | 1.02 (0.89, 1.17) | 1.01 (0.88, 1.16) |
|  | **Quintile 4** | 46,362 | 434 | 9.36 | 1.06 (0.92, 1.23) | 1.07 (0.93, 1.24) |
|  | **Quintile 5** | 39,778 | 350 | 8.80 | 1 (Ref) | 1 (Ref) |
|  | **Per 1 standard deviation decrease in Mg** | | |  | 1.07 (1.02, 1.13) | 1.04 (0.99, 1.10) |
| **Black** |  |  |  |  |  |  |
|  | **Quintile 1** | 13,648 | 170 | 12.46 | 1.33 (1.02, 1.72) | 1.29 (0.99, 1.68) |
|  | **Quintile 2** | 13,951 | 168 | 12.04 | 1.16 (0.90, 1.50) | 1.14 (0.87, 1.48) |
|  | **Quintile 3** | 14,881 | 179 | 12.03 | 1.05 (0.81, 1.35) | 1.07 (0.82, 1.38) |
|  | **Quintile 4** | 10,109 | 125 | 12.37 | 1.10 (0.84, 1.45) | 1.12 (0.85, 1.47) |
|  | **Quintile 5** | 7,411 | 87 | 11.74 | 1 (Ref) | 1 (Ref) |
|  | **Per 1 standard deviation decrease in Mg** | | |  | 1.12 (1.04, 1.21) | 1.10 (1.02, 1.19) |
| **Magnesium-race interaction***** | | | P = 0.51 | | | |

‡Crude incidence rate, per 1000 person-years.

*Adjusted for age, center of attendance, sex, and education.

**Adjusted for Model 1 variables, plus history of smoking, drinking status, waist-to-hip ratio, western and prudent diet scores, estimated glomerular filtration rate, c-reactive protein, sodium, potassium, calcium, prevalent coronary heart disease, previous stroke, systolic and diastolic blood pressure, antihypertensive diuretic medication use, total-cholesterol-to-HDL cholesterol ratio, diabetes status, and apolipoprotein E4 allele.

***Wald chi-square.
