# Supplementary material for: Low Serum Magnesium is Associated with Incident Dementia in the ARIC-NCS Cohort
Source: Nutrients. 2020 Oct 9;12(10):3074. doi: 10.3390/nu12103074 (PMC7600951; doi:10.3390/nu12103074)
Supplement: Supplementary file 1 [file nutrients-12-03074-s001.zip › Supplemental Table S3.docx]

**Supplemental Table 3. Association of baseline magnesium quintiles with incident dementia, stratified by sex, ARIC 1990-2019.**

| **Sex** | **Quintile of Magnesium** | **Person Years of Follow-Up** | | **Number developing dementia** | **IR‡** | **Model 1* HR** | **Model 2** HR** |
| --- | --- | --- | --- | --- | --- | --- | --- |
| **Male** |  |  | |  |  |  |  |
|  | **Quintile 1** | 11,237 | | 113 | 10.06 | 1.31 (1.03, 1.67) | 1.17 (0.91, 1.49) |
|  | **Quintile 2** | 19,920 | | 194 | 9.74 | 1.15 (0.94, 1.42) | 1.09 (0.89, 1.34) |
|  | **Quintile 3** | 29,068 | | 276 | 9.49 | 1.01 (0.84, 1.22) | 0.98 (0.81, 1.18) |
|  | **Quintile 4** | 25,032 | | 228 | 9.11 | 1.01 (0.83, 1.23) | 0.99 (0.81, 1.20) |
|  | **Quintile 5** | 21,163 | | 182 | 8.60 | 1 (Ref) | 1 (Ref) |
|  | **Per 1 standard deviation decrease in Mg** | | | |  | 1.10 (1.03, 1.18) | 1.07 (1.00, 1.14) |
| **Female** |  |  | |  |  |  |  |
|  | **Quintile 1** | 21,069 | | 254 | 12.06 | 1.36 (1.14, 1.62) | 1.30 (1.08, 1.56) |
|  | **Quintile 2** | 29,588 | | 281 | 9.50 | 1.08 (0.91, 1.28) | 1.07 (0.90, 1.28) |
|  | **Quintile 3** | 41,685 | | 405 | 9.72 | 1.04 (0.89, 1.21) | 1.04 (0.89, 1.22) |
|  | **Quintile 4** | 31,439 | | 331 | 10.53 | 1.12 (0.95, 1.32) | 1.13 (0.96, 1.33) |
|  | **Quintile 5** | 26,025 | | 255 | 9.80 | 1 (Ref) | 1 (Ref) |
|  | **Per 1 standard deviation decrease in Mg** | | | |  | 1.09 (1.03, 1.15) | 1.07 (1.01, 1.13) |
| **Magnesium-sex interaction***** | | | P = 0.46 | | | | |

‡Crude incidence rate, per 1000 person-years.

*Adjusted for age, race-center, and education.

**Adjusted for Model 1 variables, plus history of smoking, drinking status, waist-to-hip ratio, western and prudent diet scores, estimated glomerular filtration rate, c-reactive protein, sodium, potassium, calcium, prevalent coronary heart disease, previous stroke, systolic and diastolic blood pressure, antihypertensive diuretic medication use, total-cholesterol-to-HDL cholesterol ratio, diabetes status, and apolipoprotein E4 allele.

***Wald chi-square.
