# Supplementary material for: Low Serum Magnesium is Associated with Incident Dementia in the ARIC-NCS Cohort
Source: Nutrients. 2020 Oct 9;12(10):3074. doi: 10.3390/nu12103074 (PMC7600951; doi:10.3390/nu12103074)
Supplement: Supplementary file 1 [file nutrients-12-03074-s001.zip › Supplemental Table S4.docx]

**Supplemental Table 4. Difference in cognitive functioning at visit 2 by baseline magnesium quintile, ARIC 1990-92.**

|  | **Quintile** | **Model 1*** | **Model 2**** |
| --- | --- | --- | --- |
| **Global** |  |  |  |
|  | 1 | -0.061 (-0.099, -0.023) | -0.042 (-0.081, -0.004) |
|  | 2 | -0.035 (-0.069, -0.001) | -0.031 (-0.064, 0.003) |
|  | 3 | -0.015 (-0.046, 0.016) | -0.020 (-0.051, 0.011) |
|  | 4 | -0.002 (-0.034, 0.31) | -0.008 (-0.040, 0.024) |
|  | 5 | Reference | Reference |
| **DWRT** |  |  |  |
|  | 1 | -0.033 (-0.094, 0.028) | -0.010 (-0.072, 0.053) |
|  | 2 | -0.039 (-0.093, 0.014) | -0.032 (-0.086, 0.023) |
|  | 3 | -0.027 (-0.077, 0.022) | -0.031 (-0.080, 0.019) |
|  | 4 | 0.024 (-0.028, 0.076) | 0.019 (-0.033, 0.071) |
|  | 5 | Reference | Reference |
| **DSST** |  |  |  |
|  | 1 | -0.080 (-0.124, -0.035) | -0.049 (-0.094, -0.004) |
|  | 2 | -0.038 (-0.077, 0.002) | -0.027 (-0.066, 0.012) |
|  | 3 | -0.006 (-0.042, 0.031) | -0.006 (-0.042, 0.030) |
|  | 4 | -0.005 (-0.044, 0.034) | -0.012 (-0.050, 0.027) |
|  | 5 | Reference | Reference |
| **WFT** |  |  |  |
|  | 1 | -0.071 (-0.127, -0.015) | -0.068 (-0.125, -0.010) |
|  | 2 | -0.033 (-0.085, 0.018) | -0.038 (-0.090, 0.013) |
|  | 3 | -0.014 (-0.061, 0.034) | -0.022 (-0.070, 0.025) |
|  | 4 | -0.030 (-0.080, 0.019) | -0.039 (-0.088, 0.010) |
|  | 5 | Reference | Reference |

DWRT, delayed word recall test; DSST, digit symbol substitution test; WFT, word fluency test.

* Results from Generalized Estimating Equations adjusted for age, race-center, sex, and education.

**Adjusted for Model 1, plus history of smoking, drinking status, waist-to-hip ratio, western and prudent diet scores, estimated glomerular filtration rate, c-reactive protein, sodium, potassium, calcium, prevalent coronary heart disease, previous stroke, systolic and diastolic blood pressure, antihypertensive diuretic medication use, total-cholesterol-to-HDL cholesterol ratio, diabetes status, and apolipoprotein E4 allele.
