# Supplementary material for: Low Serum Magnesium is Associated with Incident Dementia in the ARIC-NCS Cohort
Source: Nutrients. 2020 Oct 9;12(10):3074. doi: 10.3390/nu12103074 (PMC7600951; doi:10.3390/nu12103074)
Supplement: Supplementary file 1 [file nutrients-12-03074-s001.zip › Supplemental Table S5.docx]

**Supplemental Table 5. Cognitive change over 27 years by baseline magnesium quintile, excluding the bottom 5% of test scores at baseline, ARIC 1990-2019.**

| **Test Z-Score** | **Quintiles** | **Model 1*** | **Model 2**** |
| --- | --- | --- | --- |
| **Global** |  |  |  |
|  | 1 | -0.031 (-0.094, 0.031) | 0.006 (-0.059, 0.071) |
|  | 2 | 0.041 (-0.005, 0.087) | 0.054 (0.011, 0.098) |
|  | 3 | 0.016 (-0.025, 0.056) | 0.020 (-0.019, 0.059) |
|  | 4 | -0.020 (-0.064, 0.024) | -0.021 (-0.063, 0.021) |
|  | 5 | 0 (Referent) | 0 (Referent) |
| **Per 1-SD decrease in Mg***** | | 0.001 (-0.016, 0.018) | -0.004 (-0.025, 0.016) |
| **DWRT** |  |  |  |
|  | 1 | -0.016 (-0.127, 0.095) | -0.016 (-0.105, 0.138) |
|  | 2 | 0.044 (-0.048, 0.136) | -0.056 (-0.036, 0.148) |
|  | 3 | 0.038 (-0.048, 0.125) | 0.041 (-0.046, 0.128) |
|  | 4 | -0.040 (-0.128, 0.048) | -0.044 (-0.131, 0.043) |
|  | 5 | 0 (Referent) | 0 (Referent) |
| **Per 1-SD decrease in Mg** | | 0.008 (-0.021, 0.038) | 0.007 (-0.030, 0.045) |
| **DSST** |  |  |  |
|  | 1 | -0.039 (-0.091, 0.012) | 0.003 (-0.051, 0.056) |
|  | 2 | 0.044 (0.006, 0.081) | 0.059 (0.022, 0.096) |
|  | 3 | 0.020 (-0.017, 0.057) | 0.026 (-0.011, 0.064) |
|  | 4 | -0.025 (-0.063, 0.014) | -0.024 (-0.062, 0.014) |
|  | 5 | 0 (Referent) | 0 (Referent) |
| **Per 1-SD decrease in Mg** | | 0.001 (-0.012, 0.014) | -0.009 (-0.026, 0.007) |
| **WFT** |  |  |  |
|  | 1 | -0.022 (-0.081, 0.038) | 0.014 (-0.047, 0.075) |
|  | 2 | 0.028 (-0.024, 0.080) | 0.040 (-0.013, 0.094) |
|  | 3 | -0.003 (-0.053, 0.048) | -0.0002 (-0.051, 0.050) |
|  | 4 | 0.006 (-0.043, 0.056) | 0.006 (-0.044, 0.056) |
|  | 5 | 0 (Referent) | 0 (Referent) |
| **Per 1-SD decrease in Mg** | | -0.003 (-0.019, 0.013) | -0.009 (-0.029, 0.010) |

SD, standard deviation; DWRT, delayed word recall test; DSST, digit symbol substitution test; WFT, word fluency test.

* Results from Generalized Estimating Equations adjusted for age, race-center, sex, education, and interactions of all covariates with time. Time modeled as spline term with knots at 6 years and 21 years.

**Adjusted for Model 1 variables, plus history of smoking, drinking status, waist-to-hip ratio, western and prudent diet scores, estimated glomerular filtration rate, c-reactive protein, sodium, potassium, calcium, prevalent coronary heart disease, previous stroke, systolic and diastolic blood pressure, antihypertensive diuretic medication use, total-cholesterol-to-HDL cholesterol ratio, diabetes status, apolipoprotein E4 allele, and interactions of all covariates with time. Time modeled as spline terms with knots at 6 years and 21 years.

*** SD being equal to ~0.16 mg/dL.
